# Middle Molecular Uremic Toxin and Blood Purification Therapy

**DOI:** 10.3390/jcm13030647

**Published:** 2024-01-23

**Authors:** Hideki Kawanishi

**Affiliations:** Tsuchiya General Hospital, 3-30 Nakajima-cho Naka-ku, Hiroshima 730-8655, Japan; h-kawanishi@tsuchiya-hp.jp; Tel.: +81-82-243-9191

**Keywords:** blood purification, middle molecules, hemodiafiltration, MCO-membrane, α1-microglobulin

## Abstract

The purpose of blood purification therapy is to remove uremic toxins, and middle molecules (MMs) are a specific target. An MM is defined as a solute that passes through the glomerulus with a molecular weight in the range of 0.5–58 kDa, and new classifications of “small-middle 0.5–15 kDa,” “medium-middle 15–25 kDa,” and “large-middle 25–58 kDa” were proposed. In Japan, the removal of α1-microglobulin (αMG) in the large-middle range has been the focus, but a new theory of removal has been developed, emphasizing the antioxidant effect of αMG as a physiological function. Clinical proof of this mechanism will lead to further development of blood purification therapies.

## 1. Introduction

The objective of blood purification in the treatment of patients with kidney failure or end-stage renal disease is to eliminate uremic toxins from the blood. Chronic dialysis therapy, the primary treatment for kidney failure, was pioneered in the 1960s by the Scribner Group in Seattle [1]. Uremic toxins are waste products that accumulate in the body when the kidneys can no longer effectively filter and excrete them. Various techniques, including hemodialysis (HD) and the removal of uremic toxins, have been developed to achieve this goal. Online hemodiafiltration (HDF), an advanced blood purification technique, was introduced by Henderson et al. [2]. However, the clinical application of online HDF has been limited in the U.S. [3] and has predominantly advanced in Europe [4,5]. Recently, there has been a growing number of patients in Japan and other Asian countries opting for this treatment [5].

The definition of uremic toxins has been a subject of debate since the 1970s [6]. In 2003, the EUROPEAN UREMIC TOXIN WORK GROUP (EUTOX), led by Vanholder et al., provided a comprehensive definition and classification [7]. According to them, solutes were categorized as free water-soluble low molecular weight solutes (small solutes), middle molecules, and protein-bound solutes. Additionally, they assessed the level of evidence for these uremic toxins [8]. Dialysis therapy, based on membrane separation technology, targets small to middle molecules. However, middle molecules (MMs), with molecular weights ranging from 0.5 to 58 kD, are widely distributed, and a diverse array of target substances has caused some confusion, including in terminology. A new classification was proposed in 2021 to clarify middle molecules [9].

This review aims to present the current status of blood purification therapy based on the recently proposed classification of MMs and discuss future targeted removal therapy.

### Classification of Middle Molecular Uremic Toxin

The emphasis on middle molecule (MM) removal in the context of blood purification began with a statement made by Scribner at the American Society for Artificial Organs (ASAIO) in 1965 [10]. Scribner noted that “the patients feel better on less dialysis, there is a chance that because the peritoneal membrane is leaky, we are removing with peritoneal dialysis certain higher molecular weight substances more efficiently than with hemodialysis, …… suggesting that we need a leaky membrane for a hemodialyzer”. His observation suggested the potential importance of having a “leaky membrane” in a hemodialyzer. This led to research emphasizing MM removal, established by the “square meter-hour hypothesis” [11] and “middle molecular hypothesis” [12] by Babb.

The identification of β2-microglobulin (βMG) as a causative agent of dialysis amyloidosis by Gejyo et al. [13] prompted the development of dialyzers and dialysis techniques tailored for the removal of solutes with molecular weights exceeding 10 kDa.

As these technologies evolved, the field of MM removal expanded considerably. In a recent comprehensive review [9], MMs were operationally defined as solutes falling within the molecular weight range of 0.5–58 kDa. This contemporary framework further subdivided middle molecules into distinct categories, including “small-middle” (0.5–15 kDa), “medium-middle” (15–25 kDa), and “large-middle” (25–58 kDa). Notably, this revised classification aligns the upper limit of middle molecules (58 kDa) with the limit of glomerular filtration.

The adoption of this updated classification system presents a more precise and clinically relevant framework for guiding the selection of blood purification techniques. By aligning the upper boundary of MM with the glomerular filtration limit, it furnishes a practical and consistent reference point for refining therapeutic strategies in the management of uremic toxins and optimizing the well-being of individuals afflicted by kidney failure.

While most protein-bound uremic toxins are comprised of small molecular weight compounds (<500 Da), their toxic effects are mitigated through association with proteins [14]. Consequently, the elimination of these toxins via blood purification techniques is constrained to the free, unbound fraction. To augment the removal of protein-bound uremic toxins, it becomes imperative to disrupt the protein-binding interactions and augment the free fraction. Despite experimental attempts in this direction, the translation of these strategies into established clinical practices has yet to be realized [15]. This matter thus remains a challenge for future research and clinical implementation.

## 2. Middle Molecules and the Blood Purification Setting (Figure 1)

The removal efficiency of HDF is primarily determined by the volume of replacement fluid, although removal characteristics vary depending on the dilution method employed. Post-dilution HDF, when combined with a protein-non-leakage hemodiafilter, demonstrates efficacy in the removal of small-middle molecules such as βMG. In contrast, for the targeting of medium to larger-middle molecules (e.g., κ-free light chain (FLC) 22 kDa, λ-FLCα1 44 kDa, α1-microglobulin (αMG), 33 kDa), a protein-leakage type hemodiafilter is preferred. However, the drawback of excessive albumin leakage in post-dilution HDF arises due to the challenges in effectively separating albumin from larger-middle substances. In such instances, pre-diluted HDF, capable of discriminating between albumin and larger-middle molecules, is the preferred choice. This concept is the difference between the selection of dialysis membranes in Japan and Europe, which is shown in Table 1.

**Figure 1:**
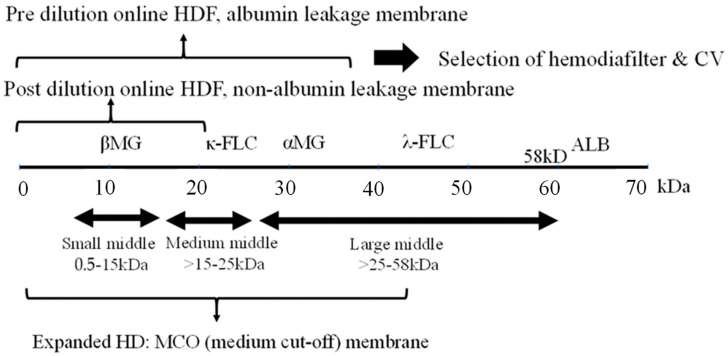
Selection of blood purification modality on middle molecules. Definition of middle molecules (MM)s: MWs 0.5 to <58 KD. CV: convection volume, βMG: β2 microglobulin, FLC: free light chain, ALB: albumin.

In Japan, pre-dilution HDF using a protein-leaking hemodiafilter is the mainstream in order to increase the removal efficiency up to αMG. However, the biological activity of αMG itself is not yet clear, and the uremic toxins in its vicinity are also unknown. On the other hand, post-dilution HDF is the mainstream in Europe to increase the removal efficiency of molecular weight up to βMG, for which evidence has been established as a uremic toxin. In addition, an increase in blood flow rate is essential to increase the convection volume (CV) in post-dilution HDF, and a blood flow rate of 300–400 mL/min is common in Europe. In Japan, the blood flow rate is less than 300 mL/min, which is another reason why pre-dilution HDF is chosen (Table 1).

As a new classification of MM, large pore membrane dialyzers have been used for the removal by HD in the medium to large-middle region [16]. Initially, a high cutoff (HCO) membrane (average pore size 10 nm) was used to remove FLC (κ- 22 kDa, λ- 44 kDa), the causative agent of renal damage due to multiple myeloma, but albumin loss was excessive and it was difficult to use in common HD.

Subsequently, medium cutoff (MCO) membranes (average pore size around 5 nm) were developed and are widely used. Terminology of expanded HD is also used in some cases to indicate that MM removal is more efficient than conventional HD. These MCO membrane dialyzers have about the same performance as protein-leakage filters (dialyzers) in Japan, which means that the super high-flux-albumin leaking dialyzer therapy in Japan is now accepted in Europe, where protein leakage has tended to be denied. Comparisons between this MCO membrane HD and post-dilution HDF have also been made [17], but at present only the name MCO precedes it and no clear criteria have been established. The new definition of MMs requires standards based on a set range of substances to be removed.

## 3. Impact of Highly Efficient Blood Purification on Patient Survival

### 3.1. Impact on HDF for Survival

The patient survival outcomes associated with HDF have been subject to extensive review [5]. The most recent investigations have focused on the survival effects of high CV achieved through post-dilution online HDF in European settings. Randomized controlled trials (RCTs), including the Dutch CONTRAST trial [18], comparing post-dilution HDF versus low flux HD, and the Turkish study [19], ESHOL study [20], and French study [21], which all compare post-dilution HDF versus high-flux HD, have collectively reported that augmenting CV with HDF (with CV values of 22 L [18], 17.2 L [19], 20.7 L [20], and 22.9–23.9 L [21] in each respective study) demonstrates a proportionate enhancement in patient survival.

However, it is essential to note that these RCTs did not explicitly establish CV as a designated treatment objective and did not definitively account for the potential presence of confounding factors. In cases where a higher CV might have been attained, such as in individuals characterized as “healthy” with fewer comorbidities, the presence of native vascular access, and higher blood flow, the risk of mortality was observed to be lower. It is worth acknowledging that these factors were not systematically controlled for in the study design.

Furthermore, the relatively low mean age of the study participants (53 years) and the absence of comprehensive information regarding the selection process and participating centers raised questions about the generalizability of the findings to the broader dialysis population. Consequently, it remained uncertain whether the procedure was universally applicable to all dialysis patients [22]. The theoretical significance of increased solute removal as a determinant of improved life expectancy also warranted further clarification, as no specific upper limit for CV had been proposed.

Conversely, an observational study conducted using Euro-DOPPS 4–5 failed to demonstrate a discernible advantage of HDF [23]. Likewise, an RCT in Australia, comparing post-dilution HDF with a CV of 24.7 L to high-flux HD, did not reveal any substantial survival benefit or improvements in neurological symptoms [24]. It is plausible that the prolonged dialysis duration (5 h) and the favorable survival rates (1-year survival exceeding 90%) in this particular study may have contributed to the absence of differential outcomes.

To address the limitations observed in prior HDF studies, two extensive RCTs were conducted in Europe [22]: the CONVINCE study (Comparison of high-dose HDF with high-flux HD) and the H4RT (High-volume HDF versus High-flux HD Registry Trial). These trials encompassed not only assessments of life expectancy but also explored outcomes related to hospitalization, quality of life, and cost-effectiveness.

In the primary analysis of CONVINCE conducted in 2023, a notable 33% reduction in all-cause mortality over a span of 3 years was reported in the high-dose HDF group (CV > 23 L) comprising 683 patients in comparison to the high-flux HD group with 677 patients. Subsequent sub-analyses highlighted specific benefits, particularly among patients without cardiovascular disease (CVD) or diabetes [25].

Nevertheless, it is essential to acknowledge that this RCT coincided with the COVID-19 pandemic, which could potentially have impacted hospitalization rates and other variables. Ongoing investigations and further analyses are slated for the future to gain a more comprehensive understanding of these findings [26].

Pre-dilution HDF in Japan is specifically tailored for αMG removal, and Sakurai et al. have reported an impressively high αMG removal rate of 35–40% or even higher, particularly for managing conditions such as bone–joint pain and restless leg syndrome [27]. The targeted removal of αMG holds significant clinical relevance, and the current objective is to establish treatment parameters that maximize αMG removal while minimizing albumin leakage. It is worth noting that a slight increase in the removal rate within the high removal rate range can lead to an excessive amount of removal [28].

Furthermore, in accordance with data analysis conducted by the Japanese Renal Data Registry (JRDR) of the Japanese Society of Dialysis Therapy (JSDT), it was observed that the survival rate improved in pre-diluted HDF with a substitution volume of 40 L or more. Notably, the substitution volume that contributed most significantly to enhanced survival was found to be 50.5 L, implying a U-shaped relationship between survival and substitution volume [29]. Additionally, Sakurai et al. demonstrated that highly efficient HDF led to the release of the platelet surface marker CD62P, suggesting increased stress on blood cell components [30].

Based on these findings, it is recommended that pre-dilution HDF in Japan be administered under conditions characterized by a blood flow rate within the range of 250–300 mL/min and a substitution volume of 200–240 mL/min (equivalent to 48–58 L).

### 3.2. Efficacy of Expanded HD

A meta-analysis examining the efficacy of expanded HD revealed superior clearance of medium to large-middle molecules, such as κ/λ FLC, in comparison to high-flux HD and post-dilution HDF. Additionally, it was associated with reduced albumin loss when contrasted with HDF [31]. However, an equivalent meta-analysis focusing on HDF did not demonstrate a survival advantage for expanded HD [32]. This underscores the continued scarcity of long-term survival evidence for expanded HD, as supported by the limited number of studies or randomized controlled trials available to date. Consequently, additional research is warranted to address this knowledge gap and provide further insights into its potential benefits.

## 4. The Functional Classification of Dialyzer

The functional classification of dialyzers in Japan was initially established by the JSDT. In 1996, it was categorized into Type I and Type II based on in vitro βMG clearance. Subsequently, in 2006, the classification was expanded to encompass Types I-IV. In 2013, further refinement was achieved with the adoption of Type Ia, Ib, IIa, and IIb classifications, which integrated in vitro albumin sieving coefficient (SC) along with βMG clearance (Table 2).

An analysis of the JRDR utilizing the 2006 classification framework revealed a significant 35% reduction in 3-year mortality among patients using Type V dialyzers when compared to Type IV dialyzers as the reference group. This result emphasizes the effectiveness of employing the so-called super high-flux membranes [33].

Conversely, in settings outside of Japan, classification typically encompassed high-flux membranes and low-flux membranes, with criteria such as βMG clearance exceeding 14 mL/min in the HEMO study [34], or conditions like βMG SC greater than 0.6 and ultrafiltration rate (UFR) exceeding 20 mL/mmHg/h in the MPO study [35] designating membranes as high-flux. The primary analysis of the HEMO study initially revealed no significant difference in mortality between the two groups; however, secondary analyses indicated a reduction in CVD mortality among long-term HD patients with a duration of more than 3.7 years [36,37]. Additionally, findings from the MPO study indicated a reduction in all-cause mortality in patients with serum albumin levels below 4.0 g/dL or those with diabetes mellitus who were treated with high-flux membranes [35]. Although these studies showed an advantage of high-flux membranes, the relationship to removed substances and the biocompatibility of dialysis membranes were not clear.

As a consequence of the development of MCO membranes and the increasing adoption of Japanese protein-leak dialyzers, predominantly in Europe since approximately 2015, the Storr classification was introduced. This classification incorporated albumin permeability alongside βMG [38]. This framework aligns with the principles outlined in the 2013 Japanese classification.

In line with this evolution, the extracorporeal blood purification meeting in November 2022 Rome introduced a classification system. This classification categorizes patients based on treatment modality (rather than membrane permeability) into low-flux HD, high-flux HD, and expanded HD, with albumin permeability serving as an additional indicator [39].

Japan has taken a leading role in the development of functional classifications for dialyzers, with Japanese dialyzers exhibiting superior performance in comparison to those from other regions [33,40]. It is of paramount importance to determine whether this enhanced performance translates into improved clinical outcomes. A prospective observational cohort study titled “Japanese study of the effects of α1-microglobulin reduction rates on survival; JAMREDS” [41] has recently been initiated. This study aims to investigate the prognosis and occurrence of cardiovascular events in dialysis patients, with the goal of elucidating the theory behind the regeneration of α1-microglobulin’s antioxidant function [42].

This review article primarily explores blood purification therapies employing membrane separation techniques. However, the limitations inherent in relying solely on membrane separation for the removal of uremic toxins necessitate the exploration of alternative strategies. Notably, in Japan, the utilization of adsorbents has been investigated for the effective removal of βMG, showcasing the potential of this complementary approach [43]. Furthermore, a proposed system combining adsorbents and membrane separation aims to address the challenges associated with the removal of large-middle molecules and protein-bound uremic toxins [44,45]. This integrated approach presents a promising avenue for overcoming the limitations of individual methods, emphasizing the need for a comprehensive solution in blood purification therapies. I look forward to further advances in blood purification technology in the future.

## 5. Conclusions

This review has examined the clinical effects of dialysis therapy based on the new classification of uremic toxins. While the efficacy of HDF is increasingly evident, uncertainties persist regarding its ability to prevent dialysis-related hypotension and associated complaints. Additionally, despite initial indications suggesting a positive impact on survival rates, these effects remain tentative. Prescriptions for blood purification, including the selection of HDF, tend to be specific to the country and facility. If the proposed efficacy of αMG removal, as advocated by Japan, is substantiated, it is anticipated that MM removal therapy will advance to a new level.

## Figures and Tables

**Table 1:** Comparison of blood purification modality between Europe and Japan.

Modality		Europe	Japan
HDF		Post-dilution, use non-albumin leakage membered	Pre-dilution, use albumin leakage membrane
	CV	20–25 L/session	48–58 L/session
	Blood flow rate	≥300 mL/min	250–300 mL/min
	Target MMs	Small middle, e.g., β2-microglobulin	Large-middle, e.g., α1-microglobulin
	Evidence	RCT: benefit to survival on higher CV	National cohort: enhanced survival was found to be substitution volume 50.5 L (limitation of CV)
Expanded HD		MCO membrane	Note: This term is not common in Japan. The 2013 functional classification II-b: super high-flux-albumin leaking membrane HD is the equivalent.
	Target MM	Medium middle, e.g., κ free light chain protein	Medium to large-middle

CV: convection volume, MMs: middle molecules, RCT: randomized controlled trial, MCO: medium cutoff.

**Table 2:** Transition of functional classification of dialyzer in Japan.

**1996**
Classified by β2-microglobulin (βMG) clearance (CL), in vitro on QB200, QD500 mL/min
Type I βMG-CL 0–10 mL/min, standard dialyzer
Type II βMG-CL >10 mL/min, high performance dialyzer:
**2006**
Classified by βMG-CL, in vitro on QB200, QD500 mL/min
Type I < βMG-CL 10 mL/min
Type II < βMG-CL 30 mL/min
Type III < βMG-CL 50 mL/min
Type IV < βMG-CL 70 mL/min
Type V ≥ βMG-CL 70 mL/min
**2013**
Classified byβMG-CL, in vitro on QB200, QD500 mL/min and abumin (alb) sieving ecoefficiency (SC), in vitro, bromocresol green (BCG) method
Type I-a βMG-CL < 70 mL/min & alb-SC < 0.03, standard high-flux (including low flux)
Type I-b βMG-CL < 70 mL/min & alb-SC ≥ 0.03, high-flux-albumin leaking
Type II-a βMG-CL ≥ 70 mL/min & alb-SC < 0.03, super high-flux
Type II-b βMG-CL ≥ 70 mL/min & alb-SC ≥ 0.03, super high-flux-albumin leaking
